# Genetic surveillance for monitoring the impact of drug use on *Plasmodium falciparum* populations

**DOI:** 10.1016/j.ijpddr.2021.07.004

**Published:** 2021-07-26

**Authors:** Yaye Die Ndiaye, Daniel L. Hartl, David McGregor, Aida Badiane, Fatou Ba Fall, Rachel F. Daniels, Dyann F. Wirth, Daouda Ndiaye, Sarah K. Volkman

**Affiliations:** a Dantec Teaching and Research Hospital, Dakar, Senegal; b Harvard University, Cambridge, MA, USA; c Harvard T.H. Chan School of Public Health, Boston, MA, USA; d Cheikh Anta Diop University, Dakar, Senegal; e Programme National de Lutte Contre le Paludisme, Senegal; f The Broad Institute, Cambridge, MA, USA; g Simmons University, Boston, MA, USA

**Keywords:** Mass drug administration (MDA), Seasonal malaria chemoprevention (SMC), Therapeutic efficacy study (TES), Population genetics, Selective sweep, Genetic surveillance

## Abstract

The use of antimalarial drugs is an effective strategy in the fight against malaria. However, selection of drug resistant parasites is a constant threat to the continued use of this approach. Antimalarial drugs are used not only to treat infections but also as part of population-level strategies to reduce malaria transmission toward elimination. While there is strong evidence that the ongoing use of antimalarial drugs increases the risk of the emergence and spread of drug-resistant parasites, it is less clear how population-level use of drug-based interventions like seasonal malaria chemoprevention (SMC) or mass drug administration (MDA) may contribute to drug resistance or loss of drug efficacy. Critical to sustained use of drug-based strategies for reducing the burden of malaria is the surveillance of population-level signals related to transmission reduction and resistance selection. Here we focus on *Plasmodium falciparum* and discuss the genetic signatures of a parasite population that are correlated with changes in transmission and related to drug pressure and resistance as a result of drug use. We review the evidence for MDA and SMC contributing to malaria burden reduction and drug resistance selection and examine the use and impact of these interventions in Senegal. Throughout we consider best strategies for ongoing surveillance of both population and resistance signals in the context of different parasite population parameters. Finally, we propose a roadmap for ongoing surveillance during population-level drug-based interventions to reduce the global malaria burden.

## Highlights

• Effective antimalarial drug use decreases *Plasmodium falciparum* genetic diversity.
• Population-level use of drug-based interventions can contribute to drug resistance.
• Calibrate drug surveillance for transmission reduction and resistance selection.
• Monitor use of seasonal malaria chemoprevention and mass drug administration.

## Graphical abstract

**Figure:**
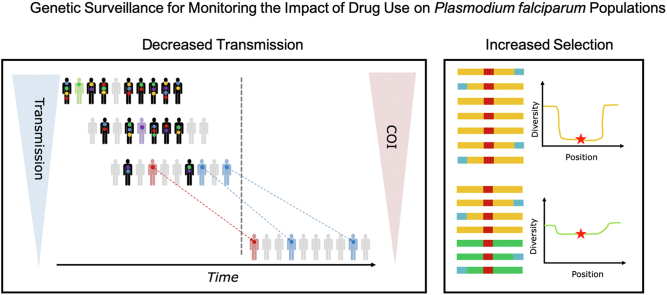

## 1 Introduction

### 1.1 Antimalarial drug use and resistance emergence

Antimalarial drugs are used both to treat infections and reduce malaria transmission. Resistance has emerged to every antimalarial drug in use, with the highest frequency of drug-resistant parasites occurring in the Greater Mekong Subregion (GMS) (van der Pluijm et al., 2021). There is evidence of drug resistance spread from the GMS to Africa (Roper et al., 2004), as well as the *de novo* emergence of drug resistance outside of the GMS (Chenet et al., 2016; Mathieu et al., 2020; Miotto et al., 2020), indicating the need for continued surveillance for drug resistance that can undermine malaria control and elimination efforts. Genetic tools can help identify resistant parasites and determine whether resistance has been introduced or emerged locally (Dalmat et al., 2019; Patel et al., 2014). Identifying the source of resistance (i.e., from spread or *de novo* emergence) is important to monitor the risk of drug resistance that may undermine drug-based interventions, and to determine strategies for controlling or slowing the development or spread of drug resistant parasites. Understanding the conditions under which drug resistance can emerge is also an important part of the effort to combat malaria. Here, we discuss the expected signatures of antimalarial drug resistance and the major factors that mitigate or contribute to this risk.

### 1.2 Monitoring drug resistance

Surveillance of drug resistance to detect and ideally track in real-time the emergence and spread of resistant malaria parasites is critical for maintaining successful malaria control and elimination efforts. Current approaches to evaluate drug resistance are accomplished by: (1) therapeutic efficacy studies (TES); (2) evaluation of parasite susceptibility to an antimalarial drug; and/or (3) monitoring validated molecular markers of drug resistance (Nsanzabana et al., 2018b). TES are prospective evaluations of the clinical and parasitological responses in patients treated for uncomplicated malaria, and provide data used by National Malaria Control Programs (NMCPs) to determine treatment policies. Parasite susceptibility to antimalarial drugs carried out *in vitro* or *ex vivo* also provides important information about parasitological response to drug exposure. Molecular surveillance of validated genetic variants that cause or contribute to antimalarial drug resistance is used to monitor the risk of drug failure and can provide data for initiating a TES, which is the ‘gold standard’ method used to monitor drug efficacy and resistance recommended by the World Health Organization (WHO) to guide drug use. While each of these strategies has advantages and disadvantages (Nsanzabana et al., 2018b), there is a clear need for robust ongoing surveillance for drug resistance. Routine surveillance has generally relied upon tracking known markers of drug resistance that are easy to monitor by simple genotyping strategies (WHO, 2020a, b); however, this approach may overlook other important indicators related to drug resistance emergence or spread, including genomic regions surrounding drug resistance loci or changes in gene copy number.

Monitoring for drug resistance historically involves the assessment of molecular markers of drug resistance, mostly single nucleotide polymorphisms (SNPs) that have been shown in laboratory settings to confer drug resistance and have been applied in the field to evaluate the emergence and spread of drug resistance. For commonly used antimalarial drugs, a few genetic variants are often surveyed (Table 1) that are localized to a limited number of genetic loci (e.g., *pfdhfr*, *pfdhps*, *pfcrt*, *pfmdr1*, *pfkelch13*) (Cowell and Winzeler, 2019; Nsanzabana et al., 2018b; WHO, 2020a). However, the use of these genetic markers alone to infer drug resistance or efficacy is limited. For example, while multiple mutations for a locus associated with drug resistance in the lab may occur at high frequency in a population (e.g., *pfdhfr*), clinical benefits are evident even in areas with reduced drug effectiveness (van Eijk et al., 2019). Drug resistance governed by some of these loci (e.g., *pfmdr1 or plasmepsin 2/3*) may involve other genetic variations such as copy number changes that are not detected by SNP analysis (Amato et al., 2017; Bopp et al., 2018; Mukherjee et al., 2017; Price et al., 2004; Qidwai, 2020; Witkowski et al., 2017). Furthermore, while drug resistance to artemisinin compounds has been shown to involve *pfkelch13* mutations (Ariey et al., 2014b), additional (Miotto et al., 2020) or alternative (Demas et al., 2018) genetic variants may be important for this resistance such that simply tracking SNPs does not fully reflect the emergence and spread of artemisinin resistance. Finally, while monitoring genetic variants may provide information about when to assess drug efficacy (WHO, 2020a), these variants have limited utility for understanding the clinical response to antimalarial drugs or for detecting novel mechanisms of drug resistance (Cowell and Winzeler, 2019; Rocamora and Winzeler, 2020; Ross and Fidock, 2019).

**Table 1:** Common *Plasmodium falciparum* drug resistance alleles monitored by genotyping.

Drug	Genetic marker
chloroquine (CQ) amodiaquine (AQ)	SNPs in *pfcrt* (K76T∗); SNPs in *pfmdr1* (N86Y∗)
piperaquine (PPQ)	SNPs in *pfcrt* (C101F, H97Y, F145I, M343 L, G353 V); Plasmepsin 2 and 3 amplifications; *pfmdr1* single copy
proguanil, pyrimethamine (PYR)	SNPss in *pfdhfr* (S108N, N51I, C59R, I164L); amplification of *gtp* cyclohydrolase 1
sulfamethoxazole, sulfadoxine	SNPs in *pfdhps* (436/437/540/581)
lumefantrine (LMF) mefloquine (MFQ)	Amplification of *pfmdr1*
Quinine	Not clear, involves mediators of LMF and MQ resistance; ms4760 microsatellites in *pfnhe-1*
Atovaquone	SNP in *cyt-b* (Y268 S/C/N)
artemisinin, artemether, DHA	SNPs in *Pfkelch13* (C580Y) (446/458/493/539/543/561) In western Thailand and Myanmar: F446I, M476I and R561H. In eastern Thailand, Cambodia, Lao People's Democratic Republic and Viet Nam): Y493H and P553L. R539T and C580Y highly prevalent in both areas. In Rwanda with increasing prevalence: R561H. In Eritrea, Ethiopia, Somalia and Sudan, and with increasing frequency in the Horn of Africa: R622I. Markers of partial resistance to artemisinin: F446I, N458Y, M476I, Y493H, R539T, I543T, P553L, R561H, P574L and C580Y.

Sources (Cowell and Winzeler, 2019; Nsanzabana et al., 2018a; WHO, 2020a).

### 1.3 Connection between drug resistance and drug efficacy

While tracking molecular markers that are associated with a change in parasite susceptibility to antimalarial drugs is useful, this strategy has limited value in predicting drug efficacy since other factors, including host immunity, can modulate drug response. The “gold standard” for evaluating drug efficacy remains the TES (WHO, 2009; 2020a); however, there are challenges and limitations in implementing this study design and interpreting the outcomes (WHO, 2020a), including a lack of standardization of genotyping approaches used to distinguish recrudescence from reinfection. Current genotyping is typically done either by nested polymerase chain reaction (PCR) of the antigen genes *msp1*, *msp2*, and *glurp* (Viriyakosol et al., 1995), or by analyzing microsatellite markers (Jones et al., 2019; WHO, 2009). Unresolved challenges to the genotyping methodology and data analysis include issues related to the classification of reinfection or treatment success (Gruenberg et al., 2019; Jones et al., 2019). Additional issues relate to parasite population structure. Low transmission settings may have less genetic diversity in the parasite population, which creates the added challenge of differentiating between recrudescent infections and reinfections of genetically similar parasites. Similarly, high-burden regions have more genetic diversity in the parasite population, which increases the risk of misclassifying reinfections and recrudescence among polygenomic infections. Evaluation and improvement of current genotyping methods and analysis tools used to evaluate drug efficacy that are appropriate for the level of transmission are greatly needed.

## 2 Drug impact on parasite population structure

### 2.1 Population effects of drug use

Challenges to evaluating the statistical and clinical impact of population-level drug-based interventions like SMC and MDA include monitoring impact on malaria burden, evaluating the clinical benefit of these interventions, and addressing whether these interventions can interrupt transmission (WHO, 2013, 2015, 2017). Historically, the impact of SMC or MDA has been assessed based on epidemiological metrics like prevalence or incidence (Access-SMC-Partnership, 2020) (Supplemental Table 1); however, these indicators may not reveal important changes to the parasite population and become more challenging to assess in certain contexts such as very low transmission settings. Measuring the impact of these interventions on clinical metrics like anemia or childhood mortality requires careful study designs that are powered to evaluate these outcomes (Cohee et al., 2020). Interruption of malaria transmission is more complicated to evaluate (McCann et al., 2020), but a desired outcome of interventions is to reduce infection and potentially disease in individuals not directly covered by the intervention. Historically, malaria transmission potential is measured by the entomological inoculation rate (EIR), the incidence of new infections, and gametocyte carriage (Tusting et al., 2014). These assessments can be challenging to obtain, however, and may benefit from models that incorporate genetic metrics of transmission (malERA-Consultative-Group, 2011; malERA-Refresh-Consultative-Panel, 2017). Genetic surveillance holds promise for monitoring population changes related to the risk of drug resistance, as well as impact evaluation of drug-based interventions on reducing malaria burden and interrupting transmission (Dalmat et al., 2019).

### 2.2 Genetic signatures of effective population impact

Genetic surveillance can detect changes in parasite population diversity over time and identify the relatedness between infections in a population to infer transmission patterns and parasite connectivity (Amambua-Ngwa et al., 2019; Auburn et al., 2012; Henden et al., 2018; Hendry J.A., 2020; Hill et al., 1995; Morgan et al., 2020; Schaffner et al., 2018; Shetty et al., 2019; Taylor et al., 2020; Taylor et al., 2019; Taylor et al., 2017; Zhu et al., 2019). Successful interventions will reduce the malaria burden and, therefore, should also reduce the genetic diversity in a parasite population. Understanding the impact of drug use on a population and whether there has been transmission interruption is critical for successful malaria elimination. Current challenges to using genetic signals to monitor transmission are identifying the specific metrics that reflect or predict changes in transmission (Daniels et al., 2015; Neafsey and Volkman, 2017; Volkman et al., 2012; Watson et al., 2021) and resolve the parasites in polygenomic infections (Chang et al., 2017; Wong et al., 2017, 2018) to better understand mechanisms of transmission. Epidemiological models are incorporating genetic information along with other epidemiological and clinical parameters to identify the specific genetic features that inform decision-making (Daniels et al., 2015; Deutsch-Feldman et al., 2020; Henden et al., 2018; Hendry J.A., 2020; Watson et al., 2021; Wesolowski et al., 2018). Technical strategies including amplicon (Aydemir et al., 2018; Boyce et al., 2018; Early et al., 2019; Juliano et al., 2016; Lerch et al., 2017; Pringle et al., 2019; Tessema et al., 2020), long-read (Imai et al., 2017, 2018; Runtuwene et al., 2018) and single-cell (Nair et al., 2014; Trevino et al., 2017) sequencing are being utilized to better assess polygenomic infections. Informatic strategies to evaluate genetic data from infections with more than one parasite present are also in development (Zhu et al., 2018, 2019). Once genetic signals have been validated, they can provide detailed information on parasite connectivity and transmission to guide intervention planning and assess the impact of interventions toward reducing malaria transmission. Validation of these models in well-designed epidemiological studies at different levels of transmission will allow a critical evaluation of genetic surveillance for decision-making. Population genetic methods provide a powerful means of both monitoring changes in the parasite population related to transmission and evaluating the potential risk of drug resistance selection on that population.

**Fig. 1:**
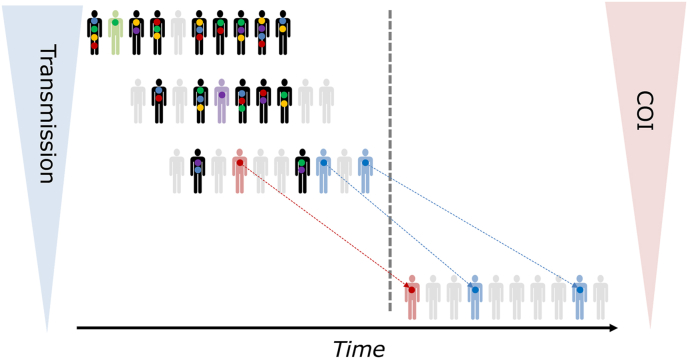
Population Genetic Indicators of Decreasing Transmission. As transmission decreases, there are fewer infected individuals (i.e., decreased incidence), represented by the silhouettes on the right with colored dots representing different parasite genotypes in the population. At high transmission levels (left), many individuals are infected, a substantial proportion of which are infected with multiple strains (black silhouettes). Elevated complexity of infection (COI) at high transmission levels decreases with reduced transmission intensity. A few individuals are infected with only one parasite type, represented by shading matching the parasite color, while a few individuals are uninfected (gray silhouettes). As transmission levels decrease, the proportion of uninfected individuals increases. In addition, more infections are monogenomic (infected with only one strain, silhouettes with only one colored dot). Finally, specific parasite types (represented by red and blue silhouettes) may be shared between transmission seasons.

Recent studies have identified genetic signatures related to decreased parasite populations, gene flow between populations, and malaria transmission indicators like *R*_0_ (Daniels et al., 2015; Hendry J.A., 2020; Watson et al., 2021). Reduction of the malaria burden will result in fewer infections, as evidenced by decreased incidence or prevalence. However, effective interventions will also reduce parasite population diversity, which is detected as a reduction in the complexity of infection (COI) or the number of different parasite genotypes found within an infected individual (Branch et al., 2001; Chang et al., 2017; Galinsky et al., 2015; Kwiek et al., 2007; Miller et al., 2017; O'Brien et al., 2016). These genetic changes arise due to increases in inbreeding as fewer parasites remain in a population, leading to an overall increase in genetic relatedness (Fig. 1). The frequency of outcrossing is also impacted by super-infection, in which multiple independent mosquito bites contribute to high COI, or co-transmission, in which multiple parasite types are transmitted by a single mosquito bite (Chang et al., 2016; Childs and Buckee, 2015; Nkhoma et al., 2012, 2018, 2020; Wong et al., 2017, 2018). The levels of transmission alter the likelihood or frequency of these different patterns of transmission, such that co-transmission of genetically similar parasites is predicted to predominate when transmission becomes low (Wong et al., 2017, 2018).

Genetic surveillance can also evaluate the relatedness between infections, as measured by increasing identity-by-descent (IBD) (Daniels et al., 2015; Henden et al., 2018; Schaffner et al., 2018; Taylor et al., 2017, 2019, 2020). These tools examine both the proportion of the genome shared between infections as well as the length of genomic stretches that are in linkage disequilibrium to provide quantitative measures of the relatedness between infections and allow inferences about transmission patterns. The appearance of clonal parasites in a population is evident when transmission levels are low and local. Several studies have observed persistent transmission of these clonal parasites between transmission seasons, indicating a reservoir of infection that may be targeted by interventions (Daniels et al., 2015, 2020). Combined with temporal (the time of sampling) and spatial (location of sampling) information, these data provide additional inferences regarding the connectivity between infections across time and space that can be used to identify both patterns and levels of transmission (Lee et al., *in preparation*). For example, measuring patterns of parasite relatedness can reveal completely distinct patterns of transmission in areas with similar epidemiological characteristics (e.g., incidence). Some settings are dominated by a clonal parasite population structure in which most parasites are nearly genetically identical. In contrast, other sites are characterized by a pattern of infections in which parasites are partially genetic related to parasites in other regions (Schaffner et al., *in preparation*). These distinct population genetic characteristics suggest very different patterns of transmission that call for appropriate intervention strategies. Thus, genetic relatedness among infections can reveal actionable information about the patterns of transmission to guide intervention selection and targeting.

## 3 Genetic signatures of drug use on the parasite population

### 3.1 Genetic signatures of drug resistance

Genetic signatures of drug pressure and drug resistance are evident as increases in the frequencies of validated drug resistant alleles and hard or soft selective sweeps of the genomic regions containing these drug-resistance variants. Tracking SNP variations alone is not sufficient for surveillance of drug resistance, as strategies targeting genomic regions that contribute to drug resistance are also important to fully understand and predict the risk of emerging resistance. When a drug-resistance mutation occurs, recurrent drug pressure gives the mutant allele a selective advantage that drives an increase in its allele frequency. In the simplest cases, the resistance is due to a SNP (Korsinczky et al., 2000) and one might naively think that the resistance gene could readily be identified by detecting a steady increase in the mutant nucleotide. This is not the case, however, as SNPs are widespread in *P. falciparum* and other malaria species and there is chance variation in their allele frequencies across time, as well as variation in their allele frequencies among subpopulations. Also, increases in allele frequencies due to drug pressure may be irregular owing merely to chance or to differences in drug administration in different geographical regions. Hence, detecting drug-resistance alleles by tracking random SNPs is usually a losing game because of noise in the genetic background due to other SNPs. Moreover, changes in allele frequency due to drug resistance affect not only the drug-resistance allele but also a region of the genome surrounding the resistance gene.

The clarity of the genetic signals associated with drug resistance depends on whether the resistance arises from standing genetic variation (pre-existing polymorphisms) or new mutations, on the nature of the resistance mutations (e.g., SNPs versus gene amplification), on the rate of new resistance mutations, on the intensity of drug pressure (i.e., the selective advantage of the resistance mutation), on the local rate of recombination in the vicinity of the resistance mutation, and on the transmission intensity. All of these also bear on the issue of surveillance.

### 3.2 Hard vs. soft selective sweeps

**Fig. 2:**
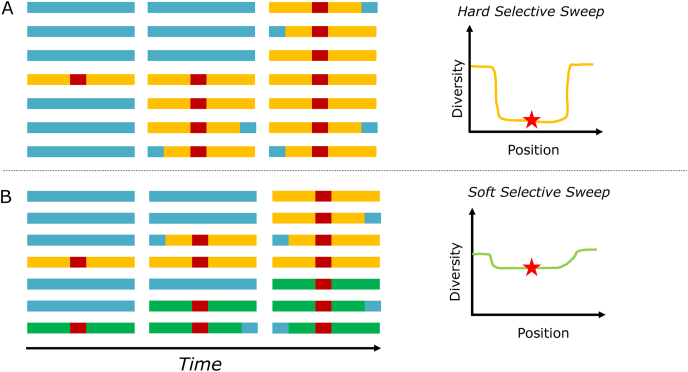
Population Genetic Signatures of Hard and Soft Selective Sweeps. (A) Hard selective sweeps result from the appearance of a new drug-resistance locus (red) on a given background haplotype (yellow) that increases in frequency. The surrounding background (yellow) will also increase in frequency in the population along with the drug-resistance allele due to genetic hitchhiking. The result is the fixation of the advantageous drug resistant allele under drug pressure. A survey of the genetic region around the advantageous allele (star) shows decreased genetic diversity that produces the pattern expected of a hard selective sweep. (B) Soft selective sweeps result when the drug-resistance allele arises on multiple backgrounds (yellow and green). Under drug pressure, the drug-resistance allele will increase in frequency; however, because there are multiple genetic backgrounds, the overall diversity in the genomic region surrounding the advantageous allele differs and there is only a small reduction in genetic diversity in the region surrounding the drug resistant allele (star).

Genetic signals of selection can differ dramatically according to whether the resistance arises from a single, rare new mutation as opposed to multiple recurring mutations or mutations that pre-exist as standing polymorphisms in the population. Selection of a single, nonrecurring mutation results in a so-called hard selective sweep (Kaplan et al., 1989; Maynard Smith and Haigh, 1974), in which a small genomic region (haplotype) around the resistance mutation increases in frequency due to drug pressure and comes to displace other haplotypes in the population. A hallmark of a hard selective sweep is the overrepresentation of a single haplotype in a population relative to what would be expected by chance (Fig. 2A). Selection of the K76T allele of PfCRT under chloroquine (CQ) pressure is an example of a hard selective sweep in malaria (Wootton et al., 2002).

The situation with multiple recurring or pre-existing mutations is very different in that each resistance mutation exists in a different haplotype; hence, multiple haplotypes increase in frequency simultaneously (Fig. 2B). This situation is known as a soft selective sweep (Hermisson and Pennings, 2005; Messer and Petrov, 2013). The detection of a soft sweep, and distinguishing it from a hard sweep, depends critically on the size and composition of the population sample (Wilson et al., 2017). A sample that is too small may reveal only one overrepresented haplotype and be misconstrued as implying a hard sweep, or it may contain multiple selected haplotypes but be insufficiently powered statistically to be distinguished from neutral standing variation. *PfKelch13* (PfK13) (Ariey et al., 2014b) has undergone a soft selective sweep (Anderson et al., 2017; MalariaGEN, 2016), and selection of different mutations including gene duplications in *pfmdr1* under mefloquine (MQ) pressure is another example of a soft selective sweep in malaria (Nair et al., 2007).

Another issue is whether the drug deployed has been used in the past (e.g., CQ or pyrimethamine [PYR]), where resistance alleles selected in the past would be part of the standing genetic variation in multiple haplotypes owing to recombination, or against a novel drug, in which case new mutations might be necessary for resistance to arise. The efficacy of a drug also matters, as there is evidence that more effective drugs result in harder selective sweeps (Cohee et al., 2020).

### 3.3 Mechanisms of drug resistance

Genetic signals of drug resistance are also affected by the nature of the underlying resistance mutations. The spectrum ranges from a unique mechanism of resistance due to mutations in a single gene to multiple mechanisms due to mutations in any of several genes. Single-gene resistance is exemplified by resistance to CQ due to amino acid replacements in PfCRT (Fidock et al., 2000), and resistance to PYR due to amino acid replacements in PfDHFR (Peterson et al., 1988; Snewin et al., 1989). In both cases the highest levels of resistance result from multiple amino acid replacements, but these are acquired sequentially (Lozovsky et al., 2009). Resistance to artemisinin is due predominantly to amino acid replacements in PfK13 (Ariey et al., 2014a), however amino acid replacements in PfCoronin can also result in artemisinin resistance *in vitro* (Demas et al., 2018).

Although new mutations resulting in specific amino acid replacements are rare, the worldwide population of *P. falciparum* is huge; thus, even single-gene resistance can have multiple origins in response to drug pressure. PfK13 resistance to artemisinin was first observed in Southeast Asia (Tun et al., 2015), but it has also arisen independently in Amazonia (Mathieu et al., 2020) and Papua New Guinea (Miotto et al., 2020). Similarly, at least six CQ resistance mutations have arisen independently in different regions of the world (Wellems et al., 2009). Although there may be multiple origins of drug resistance worldwide, within any restricted geographical region one expects only a single origin, which increases the likelihood of a hard selective sweep.

Drug resistance in *P. falciparum* can also arise via gene duplication, the classic example being resistance to MQ and halofantrine due to amplification of the multidrug resistance gene *pfmdr1* (Wilson et al., 1993). Gene duplications arise by replication slippage or ectopic recombination, and they arise readily in *P. falciparum* owing to the richness of its genome in simple-sequence repeats (Su et al., 2019). Resistance via gene duplication is, therefore, likely to have multiple origins within a single population and, hence, would result in a soft selective sweep (Nair et al., 2007).

### 3.4 Roles of recombination and transmission intensity in drug resistance

Parasite population structure, including the effective population size and opportunities for recombination, influence the selection and spread of drug-resistant parasites. Thus, the likelihood that a parasite will be exposed to antimalarial drug pressure depends upon transmission level, which reflects the parasite population size. Regions with high malaria transmission have proportionally few parasites under drug pressure, allowing recombination with parasites harboring the sensitive allele that are often more fit (Menard and Dondorp, 2017; Rosenthal, 2013) and that can re-emerge when drug pressure is withdrawn (Frosch et al., 2014; Laufer et al., 2006; Mwanza et al., 2016). Regions with low transmission (or areas where malaria control efforts have reduced transmission) have relatively few parasites that are more likely to receive sustained or repeated drug pressure. For example, CQ-resistant PfCRT haplotypes predominate in Asian populations, but the CQ-sensitive PfCRT haplotype (e.g., 3D7 type) is observed in most African parasites (Dhingra et al., 2019). Recombination plays a key role in shaping the genetic signals of resistance because the local rate of recombination determines size of the genomic region flanking the resistance allele, which shows significant linkage disequilibrium. At low transmission, inbreeding decreases the effective rate of recombination in proportion to the inbreeding coefficient, which, in principle, would increase the length of the haplotype block affected. On the other hand, at low transmission, the decrease in recombination is offset by random genetic drift due to a population bottleneck of reduced effective population size, which in itself distorts haplotype frequencies at sites across the genome.

Transmission intensity also affects genetic signals of resistance through its effect on the types of drugs used and how they are deployed. High transmission results in intermittent and reduced drug pressure, and the effect on selective sweeps depends on the tradeoff between parasite fitness in the presence versus absence of drug (Menard and Dondorp, 2017; Rosenthal, 2013). Reduced or intermittent drug pressure weakens the effective intensity of selection for resistance and prolongs the response, thereby weakening any local linkage disequilibrium and making any kind of selective sweep harder to detect. Low transmission results in more intense per-parasite drug pressure, but any signal of selection is degraded by increased inbreeding and random genetic drift.

### 3.5 Challenges and strategies for genetic surveillance of drug resistance

Surveillance for drug resistance emergence needs to employ strategies to track validated markers of resistance (typically SNPs) as well as to survey genomic regions that contribute to drug resistance. In regard to surveillance: To our knowledge, no novel drug resistance determinant has yet been identified in *P. falciparum* solely from distorted haplotype frequencies. The reasons are that, for single-gene (or a limited number of genes) resistance, signals of selection from haplotype diversity are likely to be weak for both hard selective sweeps due to population bottlenecks and increased inbreeding, and for soft sweeps due to practical limitations in sampling. Population structure also poses limitations on the power of haplotype tests. In areas of low transmission such as in Southeast Asia and South and Central America, the effective population sizes are relatively small and the resulting inbreeding reduces the effective rate of recombination, leading to distorted haplotype frequencies across the genome. In areas of high transmission, as in much of Africa, the rate of recombination is sufficiently high that the size of distorted haplotype blocks is short.

In practical terms, the most effective strategies for surveilling drug resistance are (1) to screen candidate genes (e.g., mutations coding for drug targets such as PfDHFR for PYR), (2) to culture resistant parasites and identify the region of interest by conventional genetic mapping with confirmation by gene editing (e.g., *PfCRT* for CQ), or (3) to select for resistance mutations *in vitro*, provisionally identify the gene via genomic sequencing with confirmation by gene editing (e.g., *PfK13*). Once the resistance gene or genes have been identified and confirmed by these methods, they can be surveilled individually through time and space by conventional methods as discussed elsewhere in this paper. Studies of linkage disequilibrium around the resistance genes, and the discernment of hard versus soft sweeps, can also be carried out after the fact.

These considerations bring us back to the beginning, in which we said that tracking SNPs for resistance is usually a losing game — which it is, unless you know which genes to track. Tracking resistance due to SNPs is straightforward and efficient, but tracking gene amplification is, at the moment, technically much more difficult and labor intensive.

## 4 Impact and consequences of population-level drug-based interventions: SMC, MDA

### 4.1 SMC overview (WHO recommendations)

SMC generally involves the administration of treatment doses of sulfadoxine-pyrimethamine (SP) plus amodiaquine (AQ) (SP-AQ) monthly in settings with seasonal malaria transmission (i.e., 3–4 months' duration), such as in Senegal and other West African countries as well as in Mozambique (WHO, 2013; WHO-MPAC, 2020). SMC is administered to children between 3 and 59 months of age, although some countries like Senegal have applied SMC to children up to 10 years of age. SMC has generally been shown to be an effective strategy in reducing malaria morbidity; however, the sustained use of SMC has a potential risk of selecting for drug-resistant parasites. Indeed, SMC is not recommended in regions with high levels of resistance to either SP or AQ; thus, monitoring drug resistance levels in areas of SMC is critical to both maintaining efficacy and reducing the risk of drug resistance emergence or spread.

### 4.2 Impact of SMC and drug resistance marker data on SMC use

SMC has been applied across West and Central Africa in malaria-endemic countries since the WHO recommended scale-up of the intervention in 2012 (WHO, 2013), with the main countries including Burkina Faso, Chad, Guinea, Mali, Niger, Nigeria, Senegal, and The Gambia, with expansion to Mozambique in evaluation (Consortium, 2021). There is general evidence that SMC is effective in reducing the episodes of *P. falciparum* malaria, with a Cochrane review (Meremikwu et al., 2012) reporting that SMC was effective in reducing the incidence of malaria by approximately 75 % in children under 6 years of age during the transmission season in areas with seasonal malaria transmission. Another study (Access-SMC-Partnership, 2020) also reported reduced mortality. Subsequent reviews concurred on the benefits of SMC (Ashley and Poespoprodjo, 2020; Gutman et al., 2017; McCann et al., 2020), with reports of prevalence reduction by 65 % and incidence reduction by 80 % following SMC (Greenwood, 2017; York, 2017). Extension of the age group to children up to 10 years of age was effective in reducing *P. falciparum* prevalence and incidence, although no difference in mortality was observed (Ndiaye et al., 2019). SMC may reduce the incidence in untreated age groups (Cisse et al., 2016), suggesting an interruption of transmission. There is some evidence to suggest that SMC can increase the prevalence of drug resistance markers for *pfdhps* and *pfdhfr* (Access-SMC-Partnership, 2020; Dieng et al., 2019; Maiga et al., 2016; Some et al., 2014; Turkiewicz et al., 2020). These data suggest that monitoring for resistance is an important consideration with repeated use of SMC.

### 4.3 MDA overview (WHO recommendations)

MDA has been used and delivered in different ways, from mass screening and treatment (MSAT), where only individuals positive for malaria are treated, to focal screening and treatment (FSAT) where household members of a positive case are all treated for malaria infections and mass drug administration (MDA) where typically dihydroartemisinin-piperaquine (DHAPQ) is applied to the entire population to reduce or, ideally, eliminate malaria infections in the population simultaneously to break transmission (WHO, 2017). The WHO currently recommends the use of MDA to (1) interrupt transmission in isolated or very low transmission settings with minimal risk of reintroduction; (2) in the GMS as a component of elimination efforts in light of the growing threat of multidrug-resistant malaria (Zuber and Takala-Harrison, 2018); (3) for epidemic control when other interventions are in place; and (4) in exceptional circumstances where the health system is overwhelmed (WHO, 2017). The use of MDA is controversial and only recommended once good access to case management, high coverage of effective vector control, and strong surveillance have been achieved and generally in low transmission settings or isolated areas where risk of reintroduction is very low (WHO-MPAC, 2019). To prevent the selection for drug resistance, MDA should use an ACT, ideally different from any ACT used to treat infections, achieve high programmatic coverage and adherence, and be implemented when malaria transmission is at its lowest in areas of limited risk of parasite reintroduction (Eisele, 2019).

### 4.4 Impact of MDA and drug resistance marker data on MDA use

While there is general evidence for the immediate impact of MDA, few studies support the long-term use of MDA to sustain decreased malaria prevalence (Poirot et al., 2013). The greatest successes were achieved in settings that were not exposed to imported parasite populations, such as small islands and isolated highland areas, which could achieve interruption of transmission (Poirot et al., 2013). Furthermore, areas of low endemicity were more likely to see transmission interruption as a result of MDA in combination with other interventions (Newby et al., 2015). The recent use of MDA in the GMS, with high levels of multidrug-resistant parasites, have also shown a reduction in malaria prevalence; however, many of these settings returned to pre-intervention levels within 1 year of MDA application (Zuber and Takala-Harrison, 2018). Thus, the general recommendation is to apply MDA under only specific circumstances toward malaria elimination efforts and only when there has been adequate coverage of other malaria interventions, particularly with regard to vector control (e.g., long-lasting insecticidal nets, LLINs). Evidence for the impact of MDA on parasite population structure was observed in the Zambia (Daniels et al., 2020), where DHAPQ was applied to a region after full coverage of other vector-based interventions such as LLINs. There is longstanding concern regarding the potential for MDA to introduce widespread drug resistance, such as observed with the use of table salt containing CQ starting in the 1950s (von Seidlein and Greenwood, 2003). However, so far there remains an absence of evidence linking direct MDA using therapeutic doses of an artemisinin-based drug combination (ACT) such as DHAPQ, with emerging drug resistance (Deng et al., 2018; Gupta et al., 2020; Landier et al., 2018).

## 5 Case example of Senegal

### 5.1 Intervention use in Senegal and impact on malaria burden

**Fig. 3:**
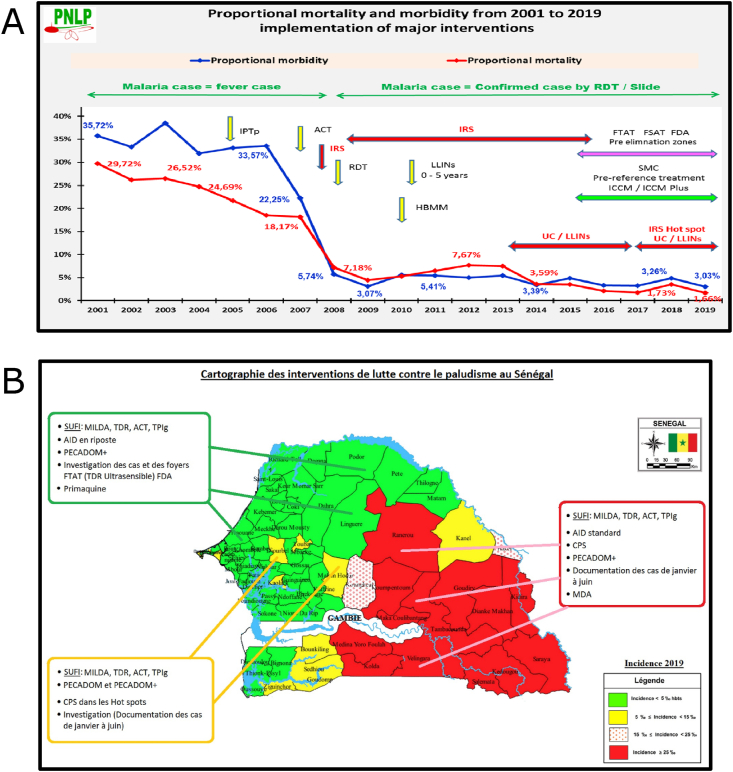
Impact and Stratification of Malaria Interventions in Senegal. (A) Proportional mortality (red line) and morbidity (blue line) between 2001 and 2019 in Senegal is shown with proportion on the y-axis and year on the x-axis. The vertical and horizontal arrows indicate the initiation and duration of various interventions, respectively. These interventions or strategies include intermittent preventive therapy in pregnancy (IPTp); artemisinin-based combination therapy (ACT); indoor residual spraying (IRS); rapid diagnostic test (RDT); long-lasting insecticide-treated nets (LLINs); home-based management of malaria (HBMM); focal test and treat (FTAT); focal screen and treat (FSAT); focal drug administration (FDA); seasonal malaria chemoprevention (SMC); integrated community case management (ICCM); and, universal coverage (UC). (B) Map showing the use of antimalarial interventions in Senegal based upon incidence: <5/1000 (green); 5/1000 to <15/1000 (yellow); 15/1000 to <25/1000 (red stippled); and ≥25/1000 (red). The interventions described in the corresponding colored boxes and abbreviations or translations include: scaled up for impact (SUFI); MILDA (long-lasting insecticide-treated nets, LLINs); AID in riposte (responsive indoor residual spraying); PECADOM (person in charge of the domicile (house); PECADOM+ (PECADOM strategy with active surveillance); case and outbreak investigation (‘investigation des cas et des foyers’); ultrasensitive RDT (TDR Ultrasensible); primaquine treatment (primaquine); AID standard (standard indoor residual spraying); seasonal malaria chemoprevention (CPS) sometimes carried out in ‘hot spots’; case documentation from January to June (‘documentation des cas de janvier a juin’); mass drug administration (MDA); intermittent preventive therapy in pregnant women (TPIg).

Senegal provides a case example of a malaria-endemic country in West Africa with a range of transmission from high (incidence > 500/1000) in the south to very low (incidence <1/1000) in the north. Senegal has made great gains over the past decade toward the reduction of malaria burden and elimination (PATH, 2018; WHO, 2020b). Overall, Senegal has achieved good coverage of interventions for malaria reduction and has implemented population-level drug-based interventions including SMC and focal MDA (PMI, 2020; PNLP, 2020). Senegal began home-based management of malaria (HBMM) and LLINs use in children aged 3–120 months in 2010. Universal coverage of LLINs began in 2013 and indoor residual spraying (IRS) for vector-based interventions was used generally between 2008 and 2015 and for targeting hotspots of transmission starting in 2017. The use of rapid diagnostic tests (RDTs) was introduced in 2008 and currently all fever cases in the country are tested with an RDT for malaria. The Integrated Community Case Management (ICCM) system started in 2015, in which malaria detection and treatment are combined with evaluation and surveillance of other diseases (Fig. 3A) (PNLP, 2020). Overall, Senegal has achieved good coverage of malaria interventions and has ongoing surveillance of malaria through its ICCM system.

Drug treatment of infections has been accomplished by ACT, beginning with artesunate amodiaquine (ASAQ) in 2006, followed by artemether-lumefantrine (AL, Coartem) in 2008, which remains the current ACT used for the treatment of malaria infections in Senegal. Population-level drug-based interventions include both intermittent preventive treatment in pregnancy (IPTp) that was introduced in 2004, and the use of SMC that was started in 2013 in districts within the Kedougou and Tambacounda regions (i.e., Dianke Makha). SMC was expanded to four regions (Kedougou, Tambacounda, Kolda, and Sedhiou) in 2014 and applied to hotspot health posts with high malaria incidence in the districts of Touba, Diourbel and Kaolack in 2019. In general, SMC involves the administration of SP-AQ in children between 3 and 120 months of age in hotspot regions where there is a high incidence of malaria. Focal drug administration strategies including FTAT (focal test and treat), FSAT (focal screen and treat), and FDA (focal drug administration) were also implemented in 2015. FTAT, FSAT, and FDA are generally implemented in regions that are classified as a pre-elimination zones based on reported incidence rates. Finally, plans are underway to use MDA in a region of higher (but still moderate) transmission to accelerate burden reduction (MESA, 2020; PMI, 2020).

The use of interventions is stratified based on reported incidence (Fig. 3B) (PNLP, 2020). In areas with low incidence, the strategies include case investigation, FDA, IPTp, LLINs, and IRS. FDA currently involves DHPAQ, with a plan to include primaquine. In regions with intermediate incidence, HBMM, IPTp, LLINs, and IRS are implemented along with SMC in children ages 3–120 months in hotspot regions identified by health facilities with high incidence. SMC is provided as three doses of SP-AQ from August to October to cover the beginning and peak of transmission. In the high incidence zone of Kedougou region that borders the Republic of Guinea, strategies include IPTp, LLINs, and IRS, with SMC provided to children aged 3–120 months in all districts. SMC is provided in four doses of SP-AQ from July to October to cover the beginning and peak of transmission.

Since adopting these strategies, Senegal has observed a dramatic decrease in proportional mortality and morbidity between 2001 and 2019, with proportional morbidity declining approximately 12-fold (from 35.72 % in 2001 to 3.03 % in 2019) and proportional mortality declining nearly 18-fold (from 29.72 % in 2001 to 1.66 % in 2019) (Fig. 3A) (PNLP, 2020). Despite these successes, it remains a challenge for the Senegal National Malaria Control Program (NMCP, which is known as the Programme National de Lutte contre le Paludisme or PNLP in Senegal) to understand and measure the impact of individual interventions on malaria burden or transmission at different levels of incidence. Ongoing work involves genetic monitoring of population changes following specific interventions as well as the evaluation of genetic indicators of drug pressure as early warning systems of drug resistance emergence and spread (PNLP, 2020).

To help accelerate these gains, Senegal will be running a two-arm, cluster-randomized controlled trial to compare the impact of MDA to standard SMC in a region of moderately high transmission (MESA, 2020). After implementing optimized malaria interventions (e.g., proactive community case management and PBO [piperonyl butoxide] nets), two sets of villages with comparable transmission levels will be included in the study. One arm of the study (30 villages) will receive SMC (SP-AQ in children 3–120 months of age); in the other arm (30 villages), three rounds of MDA (DHAPQ plus single low-dose primaquine) will be administered to the entire population except children under 6 months of age, pregnant or breastfeeding women, or individuals with other illness. The objective of the study will be to determine the effect of three rounds of MDA with DHAPQ and low-dose primaquine on village-level confirmed malaria incidence when provided in the context of optimized control interventions, compared to optimized control interventions and SMC (MESA, 2020).

### 5.2 Drug resistance surveillance in Senegal

With the increasing use of drug-based interventions, Senegal has been monitoring molecular markers of drug resistance since 2001 (PNLP, 2020) using passive case surveillance across sentinel health facilities. This surveillance includes validated molecular markers in *pfcrt*, *pfdhfr*, *pfdhps*, *pfmdr1*, and *pfk13* associated with resistance to CQ, PYR, sulfadoxine, lumefantrine, AQ, and artemisinin. The first-line antimalarial treatment switched from CQ to SP in 2001 and then to ACT in 2007. The expected changes in allele frequency, such as a decrease in the prevalence of the *pfcrt* K76T locus related to CQ resistance (Fidock et al., 2000) were observed until 2017 but has since increased. This suggests possible drug pressure on the parasite population as a result of CQ use for other medical issues like arthritis. Increased off-label use of chloroquine for COVID-19 in some settings remains an additional potential drug pressure with implications for malaria (Belayneh, 2020). SP pressure has increased with expanded SMC use in Senegal, and the prevalence of mutations in PfDHFR at loci N51I, C59R, and S108N increased along with a decrease in the A437G mutation in PfDHPS have been observed since 2016 (Dieye et al., 2016; Mbaye et al., 2017; Ndiaye et al., 2017). In 2017 and 2018, respectively, the A581G and K540E mutations in PfDHPS were first detected in Senegal in samples from areas in which SMC had been deployed. A parasite with the quintuple mutation (N51I/C59R/S108N/A437G/A581G) was identified in 2017 with the predominance of triple and quadruple mutations over subsequent years. Molecular surveillance of *pfk13* indicates the absence of mutations at amino acid positions 580, 493, 539, and 543, associated with resistance; however, the A578S mutation was observed in 2015 (Gaye et al., 2020). Finally, changes in PfMDR1 including the selection and increase in the N_86_F_184_D_1246_ haplotype associated with AL use (Okell et al., 2018) has been detected (Dieye et al., 2016; Mbaye et al., 2016), prompting discussion around rational use guidelines for AL. Senegal is performing ongoing surveillance for both individual molecular markers and haplotype classification, with evaluations of amplicon and whole genome sequencing data for both hard and soft selective sweeps.

## 6 Conclusions

Antimalarial drugs are used for both the treatment of infections and in various population-level intervention strategies, such as SMC and MDA, designed to reduce the malaria burden and potentially interrupt transmission. When possible, a consideration of which specific antimalarial drugs are optimal for clinical treatment and population-level drug-based interventions may help mitigate the risk of resistance and loss of drug efficacy. Genetic signals related to antimalarial drug use reflect their impact on parasite population structure and the potential selection for genetic variants that may contribute to increased drug resistance or decreased drug efficacy. Ongoing surveillance for both impact and resistance risk is critical to maintain the utility of these key intervention strategies for malaria control and elimination.

Effective drug use is expected to reduce genetic diversity in the parasite population, observed as decreases in COI and increases in genetic relatedness between infections that is often assessed by IBD. Measuring the impact of SMC and MDA currently relies on epidemiological indicators like decreases in prevalence or incidence that may be challenging to measure depending upon the study design and transmission context. Epidemiological modeling that incorporates genetic information about parasite population structure is critical for both identifying and assessing the sensitivity of relevant indicators to reveal informative surveillance markers of impact. However, challenges remain regarding how best to identify the key genetic features that reveal these population level signatures (Messer and Petrov, 2013). Use of laboratory studies to identify the relevant signals of resistance and their validation and informative signals from population studies will be required.

Multiple factors contribute to the selection and spread of drug-resistant parasites. These include the mechanism of resistance, the history of drug use on the population, the effective drug pressure on a population, and the transmission intensity related to the effective parasite population size. The resistance mechanisms in the malaria parasite include single point mutations, multiple mutations, and gene duplication, each of which can occur at different frequencies and require different surveillance strategies. However, monitoring the risk of resistance and potential loss of drug efficacy requires evaluation of both validated molecular markers of resistance and interrogation of the parasite genome for evidence of hard or soft selective sweeps resulting from drug pressure as an early warning system.

The so-called “arms race” (Prosser et al., 2018), the interplay between the impact of drug pressure and selection on parasite population structure, introduces programmatic challenges in that using antimalarial drugs to reduce the parasite population effectively applies more drug pressure per parasite in the population, thus increasing the risk of resistance selection. Several studies have noted that the selection of drug resistant variants or haplotypes can shape the parasite population structure. For example, evidence from the GMS suggests that repeated selection for drug-resistant variants under relatively high drug pressure has created dominant resistance lineages (Amato et al., 2018). This is also observed in high transmission contexts like the Democratic Republic of the Congo (DRC), where an east-west divide in haplotypes known to confer resistance to CQ and SP were identified along with highly related parasites over large geographic distances, indicative of gene flow and migration (Verity et al., 2020). Thus, integrated surveillance for both changes in parasite population and drug resistance metrics are essential.

Critical for the success of genomic surveillance to evaluate intervention impacts and consequences, such as drug resistance selection, is epidemiological modeling that incorporates data from well-designed studies integrating epidemiological, clinical, and molecular metrics to identify signals that predict or reflect key changes in the parasite population that can be examined in different transmission contexts. The triangulation of independent metrics to help validate these metrics for ongoing surveillance, along with the development of facile tools to allow national malaria control programs to generate, interpret, and act upon these metrics, is crucial for successful surveillance of drug impact and resistance risk.

## Declaration of competing interest

None.
