# Supplementary material for: Genetic surveillance for monitoring the impact of drug use on Plasmodium falciparum populations
Source: Int J Parasitol Drugs Drug Resist. 2021 Jul 26;17:12–22. doi: 10.1016/j.ijpddr.2021.07.004 (PMC8342550; doi:10.1016/j.ijpddr.2021.07.004)
Supplement: Multimedia component 1 [file mmc1.docx]

**Supplemental Table 1: Literature Review of Evidence of Impact and Drug Resistance Risk for SMC and MDA**

| **Authors** | **Country** | **Comparison** | **Intervention** | **Outcome** | **Comments** |
| --- | --- | --- | --- | --- | --- |
| **MDA Reviews on Impact** | | | | | |
| Eisele  (Eisele, 2019) | Zambia, Myanmar, Vietnam, Cambodia, and the Lao People’s Democratic Republic | Observational studies and randomized trials. | Single drug or multi-drug MDA. | “Effectively implemented MDA using an ACT has been shown to be safe, unrelated to the emergence of drug resistance, and may play an important role in sufficiently lowering the malaria burden to allow malaria transmission foci to be more easily identified”. |  |
| Newby (Newby et al., 2015) | 182 published accounts of MDA | Systematic, qualitative review of published, unpublished, and gray literature documenting past MDA experiences. | Importance of`:  -directly observed therapy,  -reactive monitoring (eg. adding second round of MDA if transmission persists)  -community buy-in  -co-intervention (eg. vector control) | “In general, we found that implementing MDA in higher endemicity settings will reduce transmission, but there is a much better chance of interrupting transmission when MDA is implemented in areas of low endemicity in combination with other interventions”  MDA elimination reported in isolated settings (small islands and remote villages) where points of entry are controlled and population movement is monitored.  Transmission also interrupted in large populations targeted with MDA (Tunis, Huanghuai Plain, Xinyang) | “MDA reduced parasite prevalence (or other measures of transmission) only temporarily, and transmission returned to pre-intervention levels shortly after drug administration concluded”.  MDA should be one component of a comprehensive elimination strategy including surveillance and additional measures to maintain zero or low transmission following treatment rounds. |
| Poirot et al  (Cochrane review)  (Poirot et al., 2013) | Multiple countires | Twenty-two studies (29 comparisons) compared MDA to placebo or no intervention of which two comparisons were conducted in areas of low endemicity (≤5%), 12 in areas of moderate endemicity (6-39%) and 15 in areas of high endemicity (≥ 40%). Ten studies evaluated MDA plus other vector control measures. | The studies used a wide variety of MDA regimens incorporating different drugs, dosages, timings and numbers of MDA rounds.  Two cluster-randomized trials, eight non-randomized controlled studies and 22 uncontrolled before and after studies. | “MDA appears to reduce substantially the initial risk of malaria parasitaemia. However, few studies showed sustained impact beyond six months post-MDA, and those that did were conducted on small islands or in highland settings.” |  |
| Von Seidlein and Greenwood (von Seidlein and Greenwood, 2003) | Multiple countries | Before and after intervention comparisons in 22 MDA projects. | Varied MDA regimens including: plasmoquine, proguanil, chloroquine, pyrimethamine, primaquine, sulfadoxine. | “MDAs were generally unsuccessful in interrupting transmission but, in some cases, had a marked effect on parasite prevalence and on the incidence of clinical malaria. MDAs are likely to encourage the spread of drug-resistant parasites and so have only a limited role in malaria control.” |  |
| **MDA for *P. vivax*** | | | | | |
| Rodrigues and Chaves  2019  (Marin Rodriguez and Chaves, 2019) | Costa Rica | MDA given to adults during the dry season (August and September). | Before and after intervention comparisons. | Unique dose, 7-day dosing, 14-day dosing with chloroquine + PMQ. | “malaria transmission moved from an endemic regime to a pre-elimination regime”.  “Annual number of malaria cases decreased from 2192 ± 1579 in 2000– 2009 to 18 ± 38 in 2010–2017”. |
| Phommasone  2020  (Phommasone et al., 2020) | Myanmar, Vietnam, Cambodia and Lao People’s Democratic Republic | MDA given in May, June, July 2016 to individuals older than 6 months. | Randomised controlled trials. | MDA DHAPQ + PMQ versus none | No effect on incidence and prevalence after 3 months. |
| **MDA Studies for *P. falciparum*** | | | | | |
| Daniels et al  2020  (Daniels et al., 2020) | Zambia | MDA given to 836 individuals younger than 6 years during peak malaria transmission season (April-May) 2012 and 2013, and  784 individuals of all ages above 3 months every month between December 2014 and May 2016. | Complexity of infection and parasite genotyping analysis between both groups. | Observational study of effect of population-level drug-based interventions including MDA and mass test and treatment (MTAT). | Decreased COI, increased relatedness of parasites post-MDA. |
| Eisele et al  2020  (Eisele et al., 2020) | Zambia | 271,502 individual courses of MDA given; 65,319 courses of fMDA given. Rainy season ranges from December to April. 4 rounds of MDA/fMDA:  **round 1** just before the rainy season in December 2014  **round 2** during the rainy season in February–March 2015  **round 3** during the dry season in October 2015,  **round 4** during the rainy season in February 2016. | Community-wide MDA versus household-level MDA (focal MDA). | Four rounds of DHAPQ | Decreased prevalence (31% to 4%). 18 months follow-up time after 1^st^ round of MDA/fMDA. |
| Hsiang et al  2020  (Hsiang et al., 2020) | Namibia | Passively detected confirmed index cases were eligible to trigger an intervention. Individuals residing within 500m radius of the index case were eligible to receive reactive interventions. Peak transmission season is January to June. | Reactive focal MDA only versus reactive case detection (RACD) only versus reactive focal vector control (RAVC) and RACD versus rfMDA and RCAD. | RACD: rapid diagnostic testing and treatment with artemether-lumefantrine and single-dose primaquine  rfMDA: artemether-lumefantrine  RAVC: indoor residual spraying with pirimiphos-methyl. | Primary outcome: cluster-level incidence of confirmed malaria cases 8 weeks after the first intervention.  Secondary outcome: prevalence of *P. falciparum* infection at the end of the malaria season (May to August).  Lower incidence with rfMDA compared to RACD. |
| Chaumeau et al  2019  (Chaumeau et al., 2019) | Myanmar | Four villages in Kayin State.  The rainy season usually starts in May and ends in November. | Before and after intervention. | DHAPQ + PMQ | Vivax EIR reduced 12.5 fold post MDA, but reservoir of asymptomatic carriers reconstituted within 3 months with EIR increased 5.3 fold. |
| Von Seidlein et al  2019  (von Seidlein et al., 2019) | Myanmar, Vietnam, Cambodia, and the Lao People’s Democratic Republic | MDA given to individuals in 16 villages. Transmission occurs all year but increases during the rainy season, which lasts from June to October in Myanmar, May to November in Vietnam, and May to October in Lao PDR and Cambodia. | Before and after intervention. | DHAPQ + PMQ | Reduction in prevalence and incidence of Pf over 1 year. |
| Deng et al  2018  (Deng et al., 2018) | Comoros | 97164 residents offered AP+PMQ,  224471 residents offered AP alone. 3 monthly rounds from October–December. Hyperendemic transmission season. | AP alone versus AP+PMQ | Arteminisin-piperaquine +/- PMQ | Decreased incidence of *P. falciparum* cases in both arms. |
| Landier et al  2018  (Landier et al., 2018) | Myanmar | MDA targeted at 12 465 individuals. Incidence peak in June (start of rainy season) and also December for northern study area. | Early diagnosis and treatment through malaria village posts versus targeted mass drug administration in hotspot villages. | DHAPQ MDA in hotspot villages (prevalence >40%). | Decreased incidence and prevalence of *P. falciparum* in hotspot villages. |
| Tripura et al  2018  (Tripura et al., 2018) | Cambodia | 2268 individuals targeted by MDA. Four forest-fringe villages along the Cambodian-Thai border. MDA given once a month for 3 consecutive months early in the rainy season. | MDA (intervention) in year 1 versus deferred MDA 1 year later (control) | DHAPQ | Decreased *P. falciparum* and *P. vivax* incidence. |
| Landier et al  2017  (Landier et al., 2017) | Myanmar | 3238 individuals targeted by MDA intervention. Peak incidence in July (rainy season) and January (cold season). | Pilot trial aimed to evaluate the acceptability, safety, feasibility and effectiveness of mass-drug administration (MDA) | DHAPQ + PMQ + LLINs + early detection and treatment | Decreased prevalence in intervention villages, no longer significant at 9 months post MDA. Decreased anopheline vector positivity. |
| Eisele et al  2016  (Eisele et al., 2016) | Zambia | In low transmission area, 37694 individuals targeted for MDA, 51113 individuals targeted for fMDA. In high transmission area, 45442 targeted for MDA, 47164 targeted for fMDA. Malaria transmission lasts from January to May (rainy season). | MDA versus fMDA versus no mass treatment in low and high transmission areas. | Community MDA versus household DMA with DHAPQ versus none. | Decreased incidence and prevalence in both treatment groups compared to control, more marked in high transmission areas. |
| Lwin et al  2015  (Lwin et al., 2015) | Thai-Myanmar border | 562 individuals were targeted for MDA. Peak transmission between May and December. MDA offered at monthly intervals for 3 months (June, July, and August). | Before and after MDA. | DHAPQ | *P. falciparum* prevalence and incidence reduced. |
| Noor et al  2013  (Noor et al., 2013) | Namibia (1969-1992) | From 1966 approximately 6.7 million tablets from January to June each year (2 tablets for ≥10 years old, 1 tab for < 10 years old). Main transmission season usually between March and June. | Before and after MDA. | IRS and MDA with chloroquine + pyrimethamine | Low transmission during interventions. Post intervention increased transmission and chloroquine resistance observed. |
| Song et al  2010  (Song et al., 2010) | Cambodia  17 villages in Kampong Speu province  9 villages in Kampot province | 3,653 individuals from 17 villages in Kampong targeted with MDA. 2,387 individuals in nine villages in Kampot targeted with MDA. MDA given during 6 consecutive months during the dry season. | Before and after MDA. | AP + PMQ | “Parasite rates were dramatically reduced from 52.3% to 2.6% after three years. The P. falciparum rate in children decreased from 37.0% to 1.4%, reaching 0% in eight of 17 villages. In a second field study, that included one additional mass treatment of artemisinin-piperaquine, the P. falciparum rate in children was reduced from 20.8% to 0% within six months.” |
| Von Seidlein et al  2003  (von Seidlein and Greenwood, 2003) | The Gambia | MDA occurred in June (1st to 29th). Malaria incidence is highest between September and November following the rainy season. | SP + artesunate versus placebo. | SP | No difference in incidence. |
| **SMC Reviews** | | | | | |
| Ashley and Poespoprodjo  2020  (Ashley and Poespoprodjo, 2020) | Senegal, Burkina Faso, Uganda | Children under five years targeted with SMC. | Randomized trials. | Weekly or monthly dosing. | “The risk of malaria rebounds in the seasons following mass chemoprophylaxis has been studied in several countries with inconsistent results.”  “Seasonal malaria chemoprevention is effective at preventing malaria episodes in the Sahel, Africa”  “Seasonal malaria chemoprevention has been shown to be highly effective in reducing the incidence of malaria” |
| McCann et al  2020  (McCann et al., 2020) | Mali, Burkina Faso, Senegal | Children under fiver years, and up to 10 years of age targeted with SMC. | Program evaluation (before and after intervention study), and randomised trials. | Monthly dosing during the peak transmission season. | “Several randomized clinical trials of SMC conducted in areas of West Africa with highly seasonal transmission of P. falciparum showed substantial impact on the burden of disease in children under 6 years of age” and reduced prevalence by 2/3rds in under six year olds.  “Further supportive evidence reported by studies evaluating SMC implemented through the routine health system”. |
| Gutman et al  2017  (Gutman et al., 2017) | 11 studies; Uganda, The Gambia, Senegal, Kenya, Thailand, Indonesia, Burkina Faso | Children under fiver years, children from 6-14 years, and adults targeted with SMC. | Clinical trials and a cohort study. | RCT or prospective cohort study to assess DHAPQ given as SMC or MDA (11 studies) | DHAPQ associated with decreased incidence of malaria parasitaemia. |
| Wilson et al  2011  (Wilson and Taskforce, 2011) | 12 studies; Senegal, Ghana, Mali, The Gambia, Burkina Faso. | Children under five years targeted with SMC. | Controlled and non-controlled trials. | Monthly or bimonthly dosing. | “Pooling results from twelve studies demonstrated a protective effect of IPTc against all-cause mortality of 57% (95%CI 24%-76%) during the malaria transmission season.” |
| Meremikwu et al  (Cochrane review)  2012  (Meremikwu et al., 2012) | 7 studies: Senegal, Mali, Ghana, Burkina Faso, The Gambia. | Children under six years targeted with SMC. | Individually randomized and cluster‐randomized controlled trials. | Monthly AQ + SP, monthly SP + AS, bimonthly SP. | “In areas with seasonal malaria transmission, giving antimalarial drugs to preschool children (age < 6 years) as IPTc during the malaria transmission season markedly reduces episodes of clinical malaria, including severe malaria.”  SMC prevented approximately three quarters of all clinical malaria disease episodes and three quarters of severe malaria episodes in children under 6 years of age during the transmission season. |
| **SMC Other Studies** | | | | | |
| Konaté et al  2020  (Konate et al., 2020) | Mali | Children under 5 years old targted with SMC for four months in 2015 and 2016 (July to September). | Cohort study study including all malaria clinical cases in children under five years old. In this study, 2013 and 2014 were considered as control. | SP + AQ | Significant reduction in malaria clinical incidence, both in 2015 and in 2016 of SMC implementation compared to October 2013. A slight increase of malaria incidence was observed in December at the end of SMC implementation. |
| Sacko et al  2020  (Sacko et al., 2021) | Mali | Children between three and 59 months of age targeted with SMC from August to November in 2016 and from July to October from 2017 to 2018. Peak transmission is from July to December/January. | Before and after intervention study. | SP + AQ | A decrease in incidence was observed in children under five years old in 2017 and 2018 compared to 2016 |
| Baba et al (ACCESS-SMC)  2020  (Access-SMC-Partnership, 2020) | Burkina Faso, Chad, The Gambia, Guinea, Mali, Niger, and Nigeria | Children younger than fiver years given SMC four months each year (2015 and 2016). | Case-control studies that compared receipt of SMC between patients with confirmed malaria and neighbourhood-matched community controls eligible to receive SMC. Impact on incidence and mortality was assessed from confirmed outpatient cases, hospital admissions, and deaths associated with malaria. | SP + AQ | Decreased incidence and mortality when SMC was introduced.  Molecular monitoring showed that drug resistant infections are uncommon, but some selection for resistance to sulfadoxine–pyrimethamine occurred. |
| Muhindo  2019  (Muhindo et al., 2019) | Uganda | Children from eight weeks to 24 months of age given IPT every 4 weeks or every 8 weeks. Perennial transmission. | Double-blind, randomised controlled trial of IPT with DP every 4 weeks versus every 12 weeks. | DHAPQ | “IPT with dihydroartemisinin-piperaquine given every 4 weeks was superior to treatment every 12 weeks for the prevention of malaria during childhood, and this protection was extended for up to 1 year after cessation of IPT.” |
| Ndiaye et al  2019  (Ndiaye et al., 2019) | Senegal | 24 villages, including 2,301 children aged 3–59 months and 2,245 aged 5–9 years given SMC once a month for five months starting in July 2011. Transmission season starts in June and ends in December. | SMC with community case management (CCM) (SMC villages) or CCM alone (control villages). | SP + AQ given over five months to children under 10 years of age | SMC reduced prevalence of parasitaemia. |
| Diawara et al  2017  (Diawara et al., 2017) | Mali | 1141 children aged four to 63 months targted for SMC. The transmission season is July to December. | Non-randomised before and after intervention study. One intervention district (Kita), where four rounds of SMC took place in August–November 2014, and one comparison district (Bafoulabe). | SP + AQ | “After SMC, parasitaemia prevalence fell to 18% in the intervention district and increased to 46% in the comparison district [difference-in-differences (DD) OR = 0.35; 95% CI 0.20-0.60].”  “The frequency of the quintuple mutation (dhfr N51I, C59R and S108N + dhps A437G and K540E) remained low (5%) before and after intervention in both districts.” |
| Druetz et al  2018  (Druetz et al., 2018) | Burkina Faso | Four cycles of SMC are conducted each year—once every 4 weeks (during high malaria transmission season), usually starting late July. The intervention (the first cycle of SMC) took place from July 31 through August 3, 2015. In 2014 and 2015, data collection started in mid-August and lasted about 3 weeks. 1,311 households surveyed in 2014–2015 which had at least one child aged 3–71 months. | “A pre-post study with control group was designed to measure SMC impact during high transmission season.” | SP + AQ | “SMC reduced the parasitemia point and period prevalence by 3.3 and 24% points, respectively; this translated into protective effects of 51% and 62%.” |
| Cisse et al  2016  (Cisse et al., 2016) | Senegal | Single rainy season from July to the beginning of October  transmission season (from September 15 to December 15 each year). SMC delivered once a month during the transmission seasons of 2008, 2009, and 2010 (in mid-September, mid-October, and mid-November) to children aged 3–59 months (in 2008) and to children up to 10 years of age in 2009 and 2010. | Stepped-wedge cluster-randomised design. SMC impact with children 3-59 months versus children up to 10 years of age. | SP + AQ | Decreased incidence of clinical malaria episodes in individuals older than 10 year olds decreased by 26%. |
| Bigira et al  2014  (Bigira et al., 2014) | Uganda | 393 children randomized at 6 months of age to study arm until 24 months of age.  One year additional follow-up. Year-round transmission. | No chemo-prevention versus monthly SP versus daily trimethoprim-sulfamethoxazole (TS) versus monthly DHAPQ | DHAPQ, SP, CTX, none | “Protective efficacy was 58% (95% CI, 45%-67%, p<0.001) for DP, 28% (95% CI, 7%-44%, p = 0.01) for TS, and 7% for SP (95% CI, -19% to 28%, p = 0.57)“ |
| Bojang et al  2010  (Bojang et al., 2010) | The Gambia | 1008 children under five years of age randomized for interventions at monthly intervals on three occasions during the peak malaria transmission season (September, October, and November). | Open-label, randomized trial. SP + AQ versus SP plus piperaquine (PQ) versus DHAPQ. | DP, SP + AQ, SP+PQ, none | “The incidence of malaria in the DHA plus PQ, SP plus AQ and SP plus PQ groups were 0.10 cases per child year (95% CI: 0.05, 0.22), 0.06 (95% CI: 0.022, 0.16) and 0.06 (95% CI: 0.02, 0.15) respectively. The incidence of malaria in the control group was 0.79 cases per child year (0.58, 1.08).” |
| Kamya et al  2014  (Kamya et al., 2014) | Uganda | 186 children randomized after cessation of breastfeeding and confirmed to be HIV uninfected to 24 months of age. (median 10 months of age). Year-round transmission. | Open-label, randomized controlled trial. No chemoprevention, monthly SP, daily TS, or monthly DHAPQ. | DP, SP, CTX, none | “Protective efficacy was 69% [95% confidence interval (95% CI) 53-80, P < 0.001] for dihydroartemisinin-piperaquine, 49% (95% CI 23-66, P = 0.001) for trimethoprim-sulfamethoxazole and 9% for sulfadoxine-pyrimethamine (95% CI -35 to 38, P = 0.65).” |
| Lwin et al  2012  (Lwin et al., 2012) | Thailand | 1000 adult males targeted for SMC. Transmission in the study area is “unstable, low, and seasonal”. | Randomized, Double-Blind, Placebo-Controlled Trial. | DP monthly versus DP quarterly versus placebo. Duration of study was nine months. | “The protective efficacy against all malaria at 36 weeks was 98% (95% confidence interval [CI], 96% to 99%) in the DPm group and 86% (95% CI, 81% to 90%) in the DPalt group (for both, P < 0.0001 compared to the placebo group).” |
| Nankabirwa et al  2014  (Nankabirwa et al., 2014) | Uganda | 740 children aged from 6-14 years targeted for SMC. High-intensity year-round malaria transmission. | Randomized, placebo-controlled trial DHAPQ given once a month, given every 3 months, or placebo. 12 months follow-up. | DP, DP quarterly, placebo | “IPTm reduced the incidence of malaria by 96% (95% confidence interval [CI], 88%-99%, P < .0001), the prevalence of asymptomatic parasitemia by 94% (95% CI, 92%-96%, P < .0001).” |
| Zongo et al  2015  (Zongo et al., 2015) | Burkina Faso | 1499 children under five years of age given three rounds of preventive treatment were given in August, September, and October 2009. Rainy season occurs from July to October. | Randomized noninferiority trial of DHAPQ compared with SP + AQ. | SP + AQ versus DHAPQ versus none | “The risk of a malaria attack was 0.19 in the DHAPQ group and 0.15 in the SPAQ group, an odds ratio of 1.33 (95% confidence interval [CI], 1.02 to 1.72).” |
| Somé et al  2014  (Some et al., 2014) | Burkina Faso | Children under fiver years of age targeted for SMC monthly from the end of July to the end of October. Peak transmission occurs during the rainy season (June-October). | Before and after study of 106 and 93 children respectively. | SP + AQ | “it is not yet well established whether the prophylactic failure of SMC is associated with emergence of resistance.”  “If possible, future studies should encompass multiple rounds of SMC, with testing at baseline, throughout, and after SMC to assess for the selection of resistance.” |
| Dicko et al  2011  (Dicko et al., 2011) | Mali | 3017 children aged 3–59 months taregetd for monthly treatments during peak transmission season in August, September, and October 2008. | Randomised, double-blind, placebo-controlled trial. | SP + AQ versus placebo. | “IPTc reduced the prevalence of malaria infection by 85% (95% CI 73%-92%) (p<0.001) during the intervention period and by 46% (95% CI 31%-68%) (p<0.001) at the end of the intervention period.” |
| Sesay et al  2011  (Sesay et al., 2011) | The Gambia | 1,277 children under five years of age targeted for SMC given monthly during transmission season. Peak transmission occurring during October and November. | Double-blind, randomized placebo controlled trial. | Monthly treatment with a single dose of SP + AQ versus placebo | The incidence rate of malaria in children who received IPTc was 0.44 clinical attacks per 1,000 child months at risk while that for control children was 1.32 per 1,000 child months at risk, a protective efficacy of 66%. |
| Tagbor et al  2011  (Tagbor et al., 2011) | Ghana | 1490 children aged 3– 59 months targeted for intervention from May, July and September. Transmission season is April to November. | Cluster randomised trial. | Artesunate + AQ versus none | Incidence of fevers was lower in communities given three courses of IPTc during the time of peak transmission. |
| Kweku et al  2008  (Kweku et al., 2008) | Ghana | 2451 children aged 3-59 months targeted for SMC. | Randomized, placebo-controlled trial. | placebo or artesunate + AQ monthly or bimonthly, or SP bimonthly over a period of six months. | Monthly artesunate plus amodiaquine reduced the incidence of malaria by 69% (95% CI: 63%, 74%) compared to placebo. |
| **MDA Resistance Studies** | | | | | |
| Zuber and Takala-Harrison  2018  (Zuber and Takala-Harrison, 2018) | Southeast Asia, Africa, and South America | Low and high drug resistance setting. | Observational studies. | Chloroquine MDA  Pyrimethamine MDA  DHAPQ MDA  Factors associated with emergence of drug resistance in MDA:  Drug dose, level of parasitaemia, resistance mechanism. | Evidence of emergence of resistance with MDA when subtherapeutic dose given and/or resistance appears by simple mechanism. Little evidence of increased resistance when MDA used in already high-resistance setting. |
| White  2017  (White, 2017) | Southeast Asia, South Pacific, Africa | Low and high drug resistance setting. | Observational studies. | MDA given to asymptomatic population (low parasitaemia) is unlikely to stimulate emergence of resistance.  MDA perhaps less likely to lead to emergence or resistance compared to screen-and-treat (eliminates asymptomatic carrier reservoir)  “if MDA is ineffective because of poor coverage, poor adherence, substantial migration, or surrounding high transmission, then the probability of selecting resistance might be increased. However, if MDA is effective, the probability of selecting resistance should be reduced.” | “Well conducted MDA with good adherence deployed during the dry season in areas of low seasonal transmission is likely to reduce the probability of selecting resistance.” |
| Von Seidlein and Greenwood  2003  (von Seidlein and Greenwood, 2003) | Asia, Africa | All settings | Observational studies. |  | Limited data to suggest drug resistance emergence associated with direct MDA. |
| **MDA Studies** | | | | | |
| Gupta et al  2020  (Gupta et al., 2020) | Mozambique | 1271 (November 2015) and 3752 (May 2017)  blood samples collected from random sample of individuals during and after four rounds of MDA. | During and after MDA. | DHAPQ MDA | No evidence of increased frequency of molecular markers of antimalarial resistance after MDA. |
| Rogier et al  2020  (Rogier et al., 2020) | Haiti | 757 samples analyzed for molecular drug resistance markers associated with chloroquine and SP resistance. | MDA campaigns with chloroquine/pyrimethamine in 1960s. | SP MDA | Lack of highly resistant chloroquine and SP alleles in Haiti. |
| Deng et al  2018  (Deng et al., 2018) | Comoros | 248 successful PCR products from 271 *P. falciparum*–infected blood samples collected before or after MDA. | Blood samples before or after MDA were obtained from subsets of patients who presented with symptoms and *P. falciparum* parasitemia. | Arteminisin-piperaquine +/- PMQ | No evidence for selection of PfK13. |
| Landier et al  2018  (Landier et al., 2018) | Myanmar | Over 3 years, *PfKelch13* genotypes obtained for 631 samples. | Surveillance of *PfKelch13* genotype during intervention. | DHAPQ MDA in hotspot villages (prevalence >40%) | Stable prevalence of PfK13 over study duration (3 years). |
| Landier et al  2017  (Landier et al., 2017) | Myanmar | Artemisinin resistance marker *Pfkelch13* (PfK13) genotyped in 107 *P. falciparum* infections | Before/after MDA comparisons. | DHAPQ + primaquine + LLINs + early detection and treatment | Stable prevalence of Pfk13. |
| **SMC Reviews** | | | | | |
| Turkiewicz et al  2020  (Turkiewicz et al., 2020) | 29 malaria endemic countries, notably Kenya and Tanzania. | 4,134 *P. falciparum* isolates from 2001 to 2015. | Before and after intervention study. | SMC with SP in Kenya and Tanzania | “The increased prevalence in parasites with resistant haplotypes is due to selection by drug pressure from the use of SP for IPTp and SMC”. |
| **SMC Other Studies** | | | | | |
| Dieng et al  2019  (Dieng et al., 2019) | Ghana | 535 asymptomatic schoolchildren screened across North, Central and South Ghana. | Across North, Central and South Ghana with SMC in the North in July 2015. | Children under five-years-old are given a single dose of SP combined with a 3-day course of AQ once a month for up to 4 months. | “The prevalence of pfdhfr-N51I/C59R/S108N/pfdhps-A437G quadruple mutant associated with sulfadoxine-pyrimethamine resistance was significantly higher in the north where SMC was implemented.” |
| Maiga et al  2016  (Maiga et al., 2016) | Mali | 662 and 670 children aged from three to 59 months were enrolled in the study. | Two cross-sectional surveys were conducted before (August 2012) and after (June 2014) a pilot implementation of SMC. | Children aged 3–59 months received 7 rounds of curative doses of SP + AQ over two malaria seasons. | “SMC increased the prevalence of molecular markers of P. falciparum resistance to SP in the treated children. However, there was no significant increase of these markers of resistance in the general parasite population after 2 years and 7 rounds of SMC.” |
| Somé et al  2014  (Some et al., 2014) | Burkina Faso | 120 randomly selected children aged three to 59 months from each treatment arm and an additional 120 randomly selected children from a control group that did not receive SMC. | SNP prevalence before the onset of SMC and 1 month after the third treatment. | Monthly DP or AQ + SP for 3 months in 2009 | “The prevalence of relevant mutations was increased after SMC with AQ/SP”  “In contrast to AQ/SP, SMC with DP did not clearly select for known resistance-mediating polymorphisms. SMC with AQ/SP, but not DP, may hasten the development of resistance to components of this regimen.” |
| Lo et al  2013  (Lo et al., 2013) | Senegal | Children aged less than 10 years. blood samples were obtained from 2,705 children | Genotype prevalence before and after consecutive SMC intervention. | SP + AQ once per month from September to November | “Among children with parasitaemia at the end of the transmission seasons, the CVIET haplotypes of pfcrt and the 86Y polymorphism of pfmdr1 were more common among those that had received SMC, but the number of infections detected was very low and confidence intervals were wide. The overall prevalence of these mutations was lower in SMC areas than in control areas, reflecting the lower prevalence of parasitaemia in areas where SMC was delivered.” |

SMC: seasonal malaria chemoprophylaxis; IPTc: intermittent preventive therapy for children; MDA: mass drug administration; fMDA: focal MDA; rfMDA: reactive focal MDA; AQ: amodiaquine; PMQ: primaquine; AP: artemisinin-piperaquine; SP: sulfadoxine-pyrimehtamine; DHAPQ: dihydroartemisinin-piperaquine; TS: trimethoprim-sulfamethoxazole; ACT: artemisinin-based combination therapy; MTAT: mass test and treatment; RACD: reactive case detection; RAVC: reactive focal vector control; COI: complexity of infection; IRS: indoor residual spraying; CCM: community case management; SNP: single nucleotide polymorphism; LLIN: log-lasting insecticidal nets.

Studies Cited:

Access-SMC-Partnership (2020). Effectiveness of seasonal malaria chemoprevention at scale in west and central Africa: an observational study. Lancet *396*, 1829-1840.

Ashley, E.A., and Poespoprodjo, J.R. (2020). Treatment and prevention of malaria in children. Lancet Child Adolesc Health *4*, 775-789.

Bigira, V., Kapisi, J., Clark, T.D., Kinara, S., Mwangwa, F., Muhindo, M.K., Osterbauer, B., Aweeka, F.T., Huang, L., Achan, J.*, et al.* (2014). Protective efficacy and safety of three antimalarial regimens for the prevention of malaria in young Ugandan children: a randomized controlled trial. PLoS Med *11*, e1001689.

Bojang, K., Akor, F., Bittaye, O., Conway, D., Bottomley, C., Milligan, P., and Greenwood, B. (2010). A randomised trial to compare the safety, tolerability and efficacy of three drug combinations for intermittent preventive treatment in children. PLoS One *5*, e11225.

Chaumeau, V., Kajeechiwa, L., Fustec, B., Landier, J., Naw Nyo, S., Nay Hsel, S., Phatharakokordbun, P., Kittiphanakun, P., Nosten, S., Thwin, M.M.*, et al.* (2019). Contribution of Asymptomatic Plasmodium Infections to the Transmission of Malaria in Kayin State, Myanmar. J Infect Dis *219*, 1499-1509.

Cisse, B., Ba, E.H., Sokhna, C., JL, N.D., Gomis, J.F., Dial, Y., Pitt, C., M, N.D., Cairns, M., Faye, E.*, et al.* (2016). Effectiveness of Seasonal Malaria Chemoprevention in Children under Ten Years of Age in Senegal: A Stepped-Wedge Cluster-Randomised Trial. PLoS Med *13*, e1002175.

Daniels, R.F., Schaffner, S.F., Bennett, A., Porter, T.R., Yukich, J.O., Mulube, C., Mambwe, B., Mwenda, M.C., Chishimba, S., Bridges, D.J.*, et al.* (2020). Evidence for Reduced Malaria Parasite Population after Application of Population-Level Antimalarial Drug Strategies in Southern Province, Zambia. Am J Trop Med Hyg *103*, 66-73.

Deng, C., Huang, B., Wang, Q., Wu, W., Zheng, S., Zhang, H., Li, D., Feng, D., Li, G., Xue, L.*, et al.* (2018). Large-scale Artemisinin-Piperaquine Mass Drug Administration With or Without Primaquine Dramatically Reduces Malaria in a Highly Endemic Region of Africa. Clin Infect Dis *67*, 1670-1676.

Diawara, F., Steinhardt, L.C., Mahamar, A., Traore, T., Kone, D.T., Diawara, H., Kamate, B., Kone, D., Diallo, M., Sadou, A.*, et al.* (2017). Measuring the impact of seasonal malaria chemoprevention as part of routine malaria control in Kita, Mali. Malar J *16*, 325.

Dicko, A., Diallo, A.I., Tembine, I., Dicko, Y., Dara, N., Sidibe, Y., Santara, G., Diawara, H., Conare, T., Djimde, A.*, et al.* (2011). Intermittent preventive treatment of malaria provides substantial protection against malaria in children already protected by an insecticide-treated bednet in Mali: a randomised, double-blind, placebo-controlled trial. PLoS Med *8*, e1000407.

Dieng, C.C., Gonzalez, L., Pestana, K., Dhikrullahi, S.B., Amoah, L.E., Afrane, Y.A., and Lo, E. (2019). Contrasting Asymptomatic and Drug Resistance Gene Prevalence of Plasmodium falciparum in Ghana: Implications on Seasonal Malaria Chemoprevention. Genes (Basel) *10*.

Druetz, T., Corneau-Tremblay, N., Millogo, T., Kouanda, S., Ly, A., Bicaba, A., and Haddad, S. (2018). Impact Evaluation of Seasonal Malaria Chemoprevention under Routine Program Implementation: A Quasi-Experimental Study in Burkina Faso. Am J Trop Med Hyg *98*, 524-533.

Eisele, T.P. (2019). Mass drug administration can be a valuable addition to the malaria elimination toolbox. Malar J *18*, 281.

Eisele, T.P., Bennett, A., Silumbe, K., Finn, T.P., Chalwe, V., Kamuliwo, M., Hamainza, B., Moonga, H., Kooma, E., Chizema Kawesha, E.*, et al.* (2016). Short-term Impact of Mass Drug Administration With Dihydroartemisinin Plus Piperaquine on Malaria in Southern Province Zambia: A Cluster-Randomized Controlled Trial. J Infect Dis *214*, 1831-1839.

Eisele, T.P., Bennett, A., Silumbe, K., Finn, T.P., Porter, T.R., Chalwe, V., Hamainza, B., Moonga, H., Kooma, E., Chizema Kawesha, E.*, et al.* (2020). Impact of Four Rounds of Mass Drug Administration with Dihydroartemisinin-Piperaquine Implemented in Southern Province, Zambia. Am J Trop Med Hyg *103*, 7-18.

Gupta, H., Galatas, B., Chidimatembue, A., Huijben, S., Cistero, P., Matambisso, G., Nhamussua, L., Simone, W., Bassat, Q., Menard, D.*, et al.* (2020). Effect of mass dihydroartemisinin-piperaquine administration in southern Mozambique on the carriage of molecular markers of antimalarial resistance. PLoS One *15*, e0240174.

Gutman, J., Kovacs, S., Dorsey, G., Stergachis, A., and Ter Kuile, F.O. (2017). Safety, tolerability, and efficacy of repeated doses of dihydroartemisinin-piperaquine for prevention and treatment of malaria: a systematic review and meta-analysis. Lancet Infect Dis *17*, 184-193.

Hsiang, M.S., Ntuku, H., Roberts, K.W., Dufour, M.K., Whittemore, B., Tambo, M., McCreesh, P., Medzihradsky, O.F., Prach, L.M., Siloka, G.*, et al.* (2020). Effectiveness of reactive focal mass drug administration and reactive focal vector control to reduce malaria transmission in the low malaria-endemic setting of Namibia: a cluster-randomised controlled, open-label, two-by-two factorial design trial. Lancet *395*, 1361-1373.

Kamya, M.R., Kapisi, J., Bigira, V., Clark, T.D., Kinara, S., Mwangwa, F., Muhindo, M.K., Kakuru, A., Aweeka, F.T., Huang, L.*, et al.* (2014). Efficacy and safety of three regimens for the prevention of malaria in young HIV-exposed Ugandan children: a randomized controlled trial. AIDS *28*, 2701-2709.

Konate, D., Diawara, S.I., Toure, M., Diakite, S.A.S., Guindo, A., Traore, K., Diarra, A., Keita, B., Thiam, S., Keita, M.*, et al.* (2020). Effect of routine seasonal malaria chemoprevention on malaria trends in children under 5 years in Dangassa, Mali. Malar J *19*, 137.

Kweku, M., Liu, D., Adjuik, M., Binka, F., Seidu, M., Greenwood, B., and Chandramohan, D. (2008). Seasonal intermittent preventive treatment for the prevention of anaemia and malaria in Ghanaian children: a randomized, placebo controlled trial. PLoS One *3*, e4000.

Landier, J., Kajeechiwa, L., Thwin, M.M., Parker, D.M., Chaumeau, V., Wiladphaingern, J., Imwong, M., Miotto, O., Patumrat, K., Duanguppama, J.*, et al.* (2017). Safety and effectiveness of mass drug administration to accelerate elimination of artemisinin-resistant falciparum malaria: A pilot trial in four villages of Eastern Myanmar. Wellcome Open Res *2*, 81.

Landier, J., Parker, D.M., Thu, A.M., Lwin, K.M., Delmas, G., Nosten, F.H., and Malaria Elimination Task Force, G. (2018). Effect of generalised access to early diagnosis and treatment and targeted mass drug administration on Plasmodium falciparum malaria in Eastern Myanmar: an observational study of a regional elimination programme. Lancet *391*, 1916-1926.

Lo, A.C., Faye, B., Ba el, H., Cisse, B., Tine, R., Abiola, A., Ndiaye, M., Ndiaye, J.L., Ndiaye, D., Sokhna, C.*, et al.* (2013). Prevalence of molecular markers of drug resistance in an area of seasonal malaria chemoprevention in children in Senegal. Malar J *12*, 137.

Lwin, K.M., Imwong, M., Suangkanarat, P., Jeeyapant, A., Vihokhern, B., Wongsaen, K., Snounou, G., Keereecharoen, L., White, N.J., and Nosten, F. (2015). Elimination of Plasmodium falciparum in an area of multi-drug resistance. Malar J *14*, 319.

Lwin, K.M., Phyo, A.P., Tarning, J., Hanpithakpong, W., Ashley, E.A., Lee, S.J., Cheah, P., Singhasivanon, P., White, N.J., Lindegardh, N.*, et al.* (2012). Randomized, double-blind, placebo-controlled trial of monthly versus bimonthly dihydroartemisinin-piperaquine chemoprevention in adults at high risk of malaria. Antimicrob Agents Chemother *56*, 1571-1577.

Maiga, H., Lasry, E., Diarra, M., Sagara, I., Bamadio, A., Traore, A., Coumare, S., Bahonan, S., Sangare, B., Dicko, Y.*, et al.* (2016). Seasonal Malaria Chemoprevention with Sulphadoxine-Pyrimethamine and Amodiaquine Selects Pfdhfr-dhps Quintuple Mutant Genotype in Mali. PLoS One *11*, e0162718.

Marin Rodriguez, R., and Chaves, L.F. (2019). Parasite Removal for Malaria Elimination in Costa Rica. Trends Parasitol *35*, 585-588.

McCann, R.S., Cohee, L.M., Goupeyou-Youmsi, J., and Laufer, M.K. (2020). Maximizing Impact: Can Interventions to Prevent Clinical Malaria Reduce Parasite Transmission? Trends Parasitol *36*, 906-913.

Meremikwu, M.M., Donegan, S., Sinclair, D., Esu, E., and Oringanje, C. (2012). Intermittent preventive treatment for malaria in children living in areas with seasonal transmission. Cochrane Database Syst Rev, CD003756.

Muhindo, M.K., Jagannathan, P., Kakuru, A., Opira, B., Olwoch, P., Okiring, J., Nalugo, N., Clark, T.D., Ruel, T., Charlebois, E.*, et al.* (2019). Intermittent preventive treatment with dihydroartemisinin-piperaquine and risk of malaria following cessation in young Ugandan children: a double-blind, randomised, controlled trial. Lancet Infect Dis *19*, 962-972.

Nankabirwa, J.I., Wandera, B., Amuge, P., Kiwanuka, N., Dorsey, G., Rosenthal, P.J., Brooker, S.J., Staedke, S.G., and Kamya, M.R. (2014). Impact of intermittent preventive treatment with dihydroartemisinin-piperaquine on malaria in Ugandan schoolchildren: a randomized, placebo-controlled trial. Clin Infect Dis *58*, 1404-1412.

Ndiaye, J.L.A., Ndiaye, Y., Ba, M.S., Faye, B., Ndiaye, M., Seck, A., Tine, R., Thior, P.M., Atwal, S., Beshir, K.*, et al.* (2019). Seasonal malaria chemoprevention combined with community case management of malaria in children under 10 years of age, over 5 months, in south-east Senegal: A cluster-randomised trial. PLoS Med *16*, e1002762.

Newby, G., Hwang, J., Koita, K., Chen, I., Greenwood, B., von Seidlein, L., Shanks, G.D., Slutsker, L., Kachur, S.P., Wegbreit, J.*, et al.* (2015). Review of mass drug administration for malaria and its operational challenges. Am J Trop Med Hyg *93*, 125-134.

Noor, A.M., Alegana, V.A., Kamwi, R.N., Hansford, C.F., Ntomwa, B., Katokele, S., and Snow, R.W. (2013). Malaria control and the intensity of Plasmodium falciparum transmission in Namibia 1969-1992. PLoS One *8*, e63350.

Phommasone, K., van Leth, F., Peto, T.J., Landier, J., Nguyen, T.N., Tripura, R., Pongvongsa, T., Lwin, K.M., Kajeechiwa, L., Thwin, M.M.*, et al.* (2020). Mass drug administrations with dihydroartemisinin-piperaquine and single low dose primaquine to eliminate Plasmodium falciparum have only a transient impact on Plasmodium vivax: Findings from randomised controlled trials. PLoS One *15*, e0228190.

Poirot, E., Skarbinski, J., Sinclair, D., Kachur, S.P., Slutsker, L., and Hwang, J. (2013). Mass drug administration for malaria. Cochrane Database Syst Rev, CD008846.

Rogier, E., Herman, C., Huber, C.S., Hamre, K.E.S., Pierre, B., Mace, K.E., Presume, J., Mondelus, G., Romilus, I., Elisme, T.*, et al.* (2020). Nationwide Monitoring for Plasmodium falciparum Drug-Resistance Alleles to Chloroquine, Sulfadoxine, and Pyrimethamine, Haiti, 2016-2017. Emerg Infect Dis *26*, 902-909.

Sacko, A., Sagara, I., Berthe, I., Diarra, M., Cissoko, M., Diarra, S.S., Coulibaly, D., Sanogo, M., and Dicko, A. (2021). Evolution of Malaria Incidence in Five Health Districts, in the Context of the Scaling Up of Seasonal Malaria Chemoprevention, 2016 to 2018, in Mali. Int J Environ Res Public Health *18*.

Sesay, S., Milligan, P., Touray, E., Sowe, M., Webb, E.L., Greenwood, B.M., and Bojang, K.A. (2011). A trial of intermittent preventive treatment and home-based management of malaria in a rural area of The Gambia. Malar J *10*, 2.

Some, A.F., Zongo, I., Compaore, Y.D., Sakande, S., Nosten, F., Ouedraogo, J.B., and Rosenthal, P.J. (2014). Selection of drug resistance-mediating Plasmodium falciparum genetic polymorphisms by seasonal malaria chemoprevention in Burkina Faso. Antimicrob Agents Chemother *58*, 3660-3665.

Song, J., Socheat, D., Tan, B., Dara, P., Deng, C., Sokunthea, S., Seila, S., Ou, F., Jian, H., and Li, G. (2010). Rapid and effective malaria control in Cambodia through mass administration of artemisinin-piperaquine. Malar J *9*, 57.

Tagbor, H., Cairns, M., Nakwa, E., Browne, E., Sarkodie, B., Counihan, H., Meek, S., and Chandramohan, D. (2011). The clinical impact of combining intermittent preventive treatment with home management of malaria in children aged below 5 years: cluster randomised trial. Trop Med Int Health *16*, 280-289.

Tripura, R., Peto, T.J., Chea, N., Chan, D., Mukaka, M., Sirithiranont, P., Dhorda, M., Promnarate, C., Imwong, M., von Seidlein, L.*, et al.* (2018). A Controlled Trial of Mass Drug Administration to Interrupt Transmission of Multidrug-Resistant Falciparum Malaria in Cambodian Villages. Clin Infect Dis *67*, 817-826.

Turkiewicz, A., Manko, E., Sutherland, C.J., Diez Benavente, E., Campino, S., and Clark, T.G. (2020). Genetic diversity of the Plasmodium falciparum GTP-cyclohydrolase 1, dihydrofolate reductase and dihydropteroate synthetase genes reveals new insights into sulfadoxine-pyrimethamine antimalarial drug resistance. PLoS Genet *16*, e1009268.

von Seidlein, L., and Greenwood, B.M. (2003). Mass administrations of antimalarial drugs. Trends Parasitol *19*, 452-460.

von Seidlein, L., Peto, T.J., Landier, J., Nguyen, T.N., Tripura, R., Phommasone, K., Pongvongsa, T., Lwin, K.M., Keereecharoen, L., Kajeechiwa, L.*, et al.* (2019). The impact of targeted malaria elimination with mass drug administrations on falciparum malaria in Southeast Asia: A cluster randomised trial. PLoS Med *16*, e1002745.

White, N.J. (2017). Does antimalarial mass drug administration increase or decrease the risk of resistance? Lancet Infect Dis *17*, e15-e20.

Wilson, A.L., and Taskforce, I.P. (2011). A systematic review and meta-analysis of the efficacy and safety of intermittent preventive treatment of malaria in children (IPTc). PLoS One *6*, e16976.

Zongo, I., Milligan, P., Compaore, Y.D., Some, A.F., Greenwood, B., Tarning, J., Rosenthal, P.J., Sutherland, C., Nosten, F., and Ouedraogo, J.B. (2015). Randomized Noninferiority Trial of Dihydroartemisinin-Piperaquine Compared with Sulfadoxine-Pyrimethamine plus Amodiaquine for Seasonal Malaria Chemoprevention in Burkina Faso. Antimicrob Agents Chemother *59*, 4387-4396.

Zuber, J.A., and Takala-Harrison, S. (2018). Multidrug-resistant malaria and the impact of mass drug administration. Infect Drug Resist *11*, 299-306.
